# Auxiliary Subunits Control Function and Subcellular Distribution of AMPA Receptor Complexes in NG2 Glia of the Developing Hippocampus

**DOI:** 10.3389/fncel.2021.669717

**Published:** 2021-06-10

**Authors:** Stefan Hardt, Dario Tascio, Stefan Passlick, Aline Timmermann, Ronald Jabs, Christian Steinhäuser, Gerald Seifert

**Affiliations:** Institute of Cellular Neurosciences, Medical Faculty, University of Bonn, Bonn, Germany

**Keywords:** NG2 glia, hippocampus, AMPA receptor, TARP, Ca^2+^ permeability, developmental regulation

## Abstract

Synaptic and axonal glutamatergic signaling to NG2 glia in white matter is critical for the cells’ differentiation and activity dependent myelination. However, in gray matter the impact of neuron-to-NG2 glia signaling is still elusive, because most of these cells keep their non-myelinating phenotype throughout live. Early in postnatal development, hippocampal NG2 glia express AMPA receptors with a significant Ca^2+^ permeability allowing for plasticity of the neuron-glia synapses, but whether this property changes by adulthood is not known. Moreover, it is unclear whether NG2 glia express auxiliary transmembrane AMPA receptor related proteins (TARPs), which modify AMPA receptor properties, including their Ca^2+^ permeability. Through combined molecular and functional analyses, here we show that hippocampal NG2 glia abundantly express TARPs γ4, γ7, and γ8 as well as cornichon (CNIH)-2. TARP γ8 undergoes profound downregulation during development. Receptors of adult NG2 glia showed an increased sensitivity to blockers of Ca^2+^ permeable AMPA receptors, but this increase mainly concerned receptors located close to the soma. Evoked synaptic currents of NG2 glia were also sensitive to blockers of Ca^2+^ permeable AMPA receptors. The presence of AMPA receptors with varying Ca^2+^ permeability during postnatal maturation may be important for the cells’ ability to sense and respond to local glutamatergic activity and for regulating process motility, differentiation, and proliferation.

## Highlights

– The Ca^2+^ permeability of AMPA receptors in NG2 glia of the hippocampus increases during postnatal maturation.
– This increase was proven by applying solutions with Ca^2+^ as the sole permeant ion as well as receptor inhibition by polyamines.
– The AMPA receptor complexes are associated with various auxiliary subunits, particularly TARPs γ4, γ7, γ8, and CNIH-2.
– The developmental increase of Ca^2+^ permeability of the receptors accompanies downregulation of TARP γ8.

## Introduction

AMPA receptors co-assemble with auxiliary subunits, called transmembrane AMPA receptor regulatory protein (TARP), which modulates receptor function. The subunit TARP γ2, also called stargazin, is necessary for surface translocation and synaptic expression of AMPA receptors as demonstrated in cerebellar granule cells (Chen et al., 2000; Tomita et al., 2005). Meanwhile a whole family of TARPs has been identified, which show region specificity in their expression and interact with AMPA receptors in various cell types (Mauric et al., 2013). Thus, in the hippocampus TARP γ4 was specifically identified in glial cells but not in neurons (Tomita et al., 2003). TARPs do not only affect translocation of the receptor channels but also influence its function such as binding affinity and efficacy, receptor desensitization and deactivation, and sensitivity to intracellular polyamine block [reviewed by Jackson and Nicoll (2011)]. In heterologous expression systems and cerebellar stellate cells, TARP expression may affect polyamine block and enhance channel conductance (Soto et al., 2007; Jackson et al., 2011). Furthermore it was suggested that TARPs may influence the Ca^2+^ permeability of AMPA receptors (Kott et al., 2009). Specifically, co-assembly with distinct TARPs determines the Ca^2+^ permeability of the receptors. In stellate cells of the cerebellum, TARP γ2 promotes synaptic expression of Ca^2+^ impermeable AMPA receptors while its deletion increased the proportion of extrasynaptic Ca^2+^ permeable AMPA receptors associated with TARP γ7 (Bats et al., 2012). In granule cells, however, synaptic localization of Ca^2+^ permeable AMPA receptors requires TARP γ7, while γ2 promotes expression of Ca^2+^ impermeable AMPA receptors at postsynapses (Studniarczyk et al., 2013). TARP γ8 is predominantly expressed in the hippocampus, striatum and amygdala, but not in the cerebellum (Tomita et al., 2003; Fukaya et al., 2005). Genetic deletion of TARP γ8 led to mislocation of AMPA receptors and impaired synaptic plasticity in hippocampal neurons (Rouach et al., 2005). The preferred expression of TARP γ8 in the hippocampus initiated the search for inhibitors of this subunit to prevent hyperexcitation as observed in temporal lobe epilepsy or anxiety disorders (Maher et al., 2017). Another family of AMPA receptor-associated proteins is formed by the cornichons (CNIHs), among which CNIH-2 and CNIH-3 are abundantly expressed in the brain and assemble with GluA subunits (Schwenk et al., 2009). CNIHs increase surface expression of AMPA receptors in neural cells and slow deactivation and desensitization of glutamate-evoked responses (Schwenk et al., 2009). CNIH-2 has been shown to modulate receptor gating and pharmacology of AMPA receptor-TARP complexes (Kato et al., 2010; Gill et al., 2011).

NG2 glial cells express functional AMPA receptors and receive direct synaptic input from glutamatergic neurons (Bergles et al., 2000; Jabs et al., 2005; Haberlandt et al., 2011). In cultured oligodendrocyte precursor cells (OPCs) from optic nerve as well as in CG4 cells, concomitant activation of metabotropic glutamate receptors (mGluRs) increases expression of Ca^2+^ permeable AMPA receptors, while activation of P2Y receptors led to a decrease (Zonouzi et al., 2011). The latter study also suggested that AMPA receptors in OPCs co-assemble with TARPs to influence expression of Ca^2+^ permeable AMPA receptors at climbing fiber-NG2 glia synapses of the cerebellum. RT-PCR data from optic nerve OPCs identified TARP subunits γ2, γ3, γ4, γ5, and γ6. Microarray analysis in PDGFRα-positive NG2 glia (OPCs) from mouse forebrain revealed abundant expression of TARP γ4, γ8 and, to a lower extent, γ5 (Cahoy et al., 2008). Expression of Ca^2+^ permeable AMPA receptors requires full length TARPS, which interact with the postsynaptic scaffold protein PSD-95 (Zonouzi et al., 2011). Bergmann glia express Ca^2+^ permeable AMPA receptors lacking GluA2 (Saab et al., 2012), which are associated with TARP γ5 to modify rectification properties and influence surface expression of the receptors (Soto et al., 2009).

NG2 glial cells in the hippocampus express AMPA receptors with an intermediate Ca^2+^ permeability (Seifert and Steinhäuser, 1995; Seifert et al., 1997b). In newborn mice, the receptors of these cells are Ca^2+^ permeable (Seifert et al., 2003). Their activation provokes intracellular Ca^2+^ elevations (Jabs et al., 1994; Haberlandt et al., 2011; Barron and Kim, 2019), which constitute the basis for plasticity of neuron-glia synapses (Ge et al., 2006). During maturation, Ca^2+^ permeability and sensitivity to intracellular polyamine block of the receptor channels decrease (Seifert et al., 2003; Ge et al., 2006). In hilar NG2 glia, amplitude and frequency of receptor-mediated synaptic currents increase (Mangin et al., 2008) while in the hippocampus, synaptic connectivity decreases in older mice (Passlick et al., 2016) and AMPA receptors are downregulated (Kukley et al., 2010).

Expression and functional consequences of TARPs in NG2 glia of the hippocampus have not yet been studied during development. Here we report that these cells abundantly express TARPs γ4, γ7, and to a lower extent γ8. In addition, the auxiliary subunit CNIH-2 was found in almost all cells analyzed. During postnatal development, expression of most TARPs and CNIH-2 was downregulated. Our functional analyses revealed that the sensitivity to polyamine block of Ca^2+^ permeable receptors increases in maturing NG2 glia. Moreover, the proportion of Ca^2+^ permeable AMPA receptors at the soma increases during development, which is accompanied by downregulation of TARP γ8.

## Materials and Methods

### Preparation of Brain Slices and Acutely Isolated Cells

Transgenic mice [NG2ki-EYFP (Karram et al., 2008)] of postnatal days (p) 6-12 (referred to as p10) and p40-76 (p60) were anaesthetized, sacrificed by decapitation and their brains were dissected, washed, and the hemispheres were cut into 300 μm thick slices in frontal or horizontal orientation using a vibratome (Leica Microsystems VT 1200S, Wetzlar, Germany). Slice preparation was performed at 6°C in ACSF supplemented with sucrose (preparation solution). Subsequently, slices were stored for at least 30 min in preparation solution at 35°C and afterward in ACSF at room temperature. ACSF was gassed with carbogen. Preparation solution contained (in mM): 87 NaCl, 2.5 KCl, 1.25 NaH_2_PO_4_, 7 MgSO_4_, 0.5 CaCl_2_, 25 glucose, 25 NaHCO_3_, and 105 sucrose (325 mOsm, pH 7.4). For acute cell isolation, slices were stored in HEPES-buffered solution lacking nominal Ca^2+^ (pH 7.4; 6°C).

### Patch Clamp-Recordings *in situ*

Membrane currents were measured with the patch-clamp technique in the whole-cell configuration. Current signals were amplified (EPC-7 or EPC 800, HEKA elektronik, Lambrecht, Germany), filtered at 3 and 10 kHz and sampled at 10 and 100 kHz by an interface connected to a computer system, which also served as a stimulus generator. The resistance of the patch pipettes was 2–4 MΩ (borosilicate glass; Hilgenberg). Capacitance compensation was used to improve voltage clamp control. The input resistance was determined by stepping the membrane from −70 to −60 mV. To block K^+^ channels, Na^+^ channels and GABA_*A*_ receptors, Ba^2+^ (100 μM), quinine (200 μM), TTX (0.5 μM) and picrotoxin (100 μM) was added to the bath solution. AMPA receptor agonists, modulators and blockers were applied *via* the bath solution. The artificial cerebrospinal fluid (aCSF) contained (in mM): 126 NaCl, 3 KCl, 1.25 NaH_2_PO_4_, 2 MgCl_2_, 2 CaCl_2_, 10 glucose, 26 NaHCO_3_ (gassed with carbogen, pH 7.4, 310 mOsm). The pipette solution was composed of (in mM): 130 KCl, 0.5 CaCl_2_, 2 MgCl_2_, 5 BAPTA, 10 HEPES, and 3 Na_2_-ATP (pH 7.28). Recordings were obtained at room temperature.

For analysis of postsynaptic currents, a CsCl-based pipette solution was used (in mM): 120 CsCl, 2 MgCl_2_, 0.5 CaCl_2_, 5 BAPTA, 10 Hepes, 3 Na_2_-ATP, 10 TEA. Stimulation of Schaffer collaterals was performed through a chlorinated silver electrode inserted in a low resistance (<1 MΩ), aCSF-filled glass capillary which served as a monopolar stimulation electrode. Pulse sequences were generated with an AM-Systems isolation pulse stimulator (model 2100, Jerusalem, Israel). The stimulation electrode was positioned close to the patch electrode (distance ∼60 μm, pulse duration 100 μs). Responses were analyzed with custom-written macros in Igor Pro 8 software (Wavemetrics). Stimulus artifacts were offline compensated by subtracting averaged failures. Recordings were obtained at 35°C.

### Recordings From Freshly Isolated Cells

Freshly isolated cells were obtained from slices after protease treatment as described (Matthias et al., 2003). K^+^ channel blockers (100 μM Ba^2+^, 100 μM quinine) as well as AMPA receptor agonists, blockers and modulators were applied by transferring the cells with a tube electrode to the different solutions (Seifert and Steinhäuser, 1995). Membrane currents were measured in the whole-cell configuration as described above. The resistance of the patch pipettes was 4 MΩ, the input resistance was determined as described above. The bath solution contained (in mM): 150 NaCl, 5 KCl, 2 MgCl_2_, 2 CaCl_2_, 10 HEPES, and 10 glucose (pH 7.4). The pipette solution was the same as for slice recordings. For recordings in high Ca^2+^ solution, 150 mM NaCl was replaced by 50 mM CaCl_2_ and adjusted with *N*-methyl-D-glucamine (NMDG) to an osmolarity of 320 mOsm (pH 7.4). As for recording synaptic currents (cf. above), CsCl-based pipette solution was used. Recordings were obtained at room temperature. Salts and buffers were purchased from AppliChem (Darmstadt, Germany), kainate and CTZ were received from Abcam (Milton, United Kingdom), Naspm from Alomone Labs (Israel), and IEM-1460, JNJ 55511118 and GYKI53655 from Tocris (Bristol, United Kingdom).

### Single-Cell RT-PCR

Single-cell transcript analysis was performed as previously reported (Seifert et al., 1997a; Matthias et al., 2003). Briefly, after slice recording the cell at the tip of the pipette was lifted above the slice and aspirated into the recording pipette under microscopic control. The cell content and ∼3 μl of the pipette solution were expelled into a reaction tube containing 3 μl of DEPC-treated water. Only single cells without any adherent tissue debris were selected for the analysis. The reaction tube was frozen and stored at –80°C until reverse transcription (RT). The RT mastermix contained first strand buffer (Invitrogen, Karlsruhe, Germany), dithiothreitol (DTT, 10 mM), dNTPs (4 μM × 250 μM; Applied Biosystems, Darmstadt, Germany), RNasin^TM^ (20 U; Promega, Mannheim, Germany), random hexamer primers (50 μM; Roche, Mannheim, Germany), and reverse transcriptase (SuperscriptIII, 100 U, Invitrogen). The reaction mix was added to the harvested cell content, final volume was 10 μl, and the reaction mix was incubated at 37°C for 1 h. A multiplex two-round single-cell PCR was performed with primers for TARPs, CNIH-2 and the housekeeping gene PDGFRα. The first PCR was performed after adding PCR buffer, MgCl_2_ (2.5 mM), dNTP (4 μM × 50 μM), primer (200 nM each), and 5 U *Taq* polymerase (Invitrogen) to the RT product (final volume 50 μl). Thirty-five cycles were performed (denaturation at 94°C, 25 s; annealing at 51°C, 2 min for the first five cycles, and 45 s for the remaining cycles; extension at 72°C, 25 s; final elongation at 72°C, 7 min). An aliquot (2 μl) of the PCR product was used as a template for the second PCR (35 cycles; annealing at 54°C, first five cycles: 2 min, remaining cycles: 45 s) using nested primers (Supplementary Table 1). The conditions were the same as described for the first round, but Platinum *Taq* polymerase (2.5 U; Invitrogen) was added. Products were identified with gel electrophoresis. As a positive control, total RNA from mouse brain was run in parallel. Negative controls were performed using distilled water or bath solution for RT-PCR.

### FAC Sorting and RT-qPCR

NG2ki-EYFP mice (p10 and p60, male and female) were sacrificed and their brains were dissected and the hippocampus was isolated under microscopic control (Stereo microscope, Zeiss, Germany). A cell suspension was prepared by mincing the tissue, digesting in papain (37°C, 15 min) and incubating with DNAseI [10 min; Neural Dissociation Kit (P), Miltenyi, Germany]. Fluorescent NG2 glial cells were identified by their EYFP fluorescence (emission at 527 nm) and sorted by a FACSAriaIII flow cytometer (70 μm nozzle, BD Biosciences, Heidelberg, Germany) into tubes containing Hanks’ balanced salt solution (HBSS, Ca^2+^- and Mg^2+^-free). After centrifugation (2,000 *g*, 10 min) the supernatant was discarded and the cells were suspended in 200 μl lysis/binding buffer (Invitrogen, Darmstadt, Germany), frozen in liquid nitrogen and stored at −80°C. Messenger RNA was isolated from isolated cells by cell lysis in the lysis/binding buffer and by using oligo(dT)25-linked Dynabeads (Invitrogen). The beads with adherent mRNA were suspended in DEPC-treated water (20 μl). For first strand synthesis, RT was performed using oligo-dT_24_-primer (5 μM, Eurogentec). The reaction mix was incubated for 1 h at 50°C (final volume 40 μl).

The reaction mixture for real-time PCR contained Takyon real-time PCR mastermix (Eurogentec, Seraing, Belgium) and Taqman primer/probe mix (Thermo Fisher Scientific, Darmstadt, Germany). One μl of the RT product was added, the reaction volume was 12.5 μl. PCRs for the respective target genes and β-actin, as a housekeeping gene, were run in parallel wells for each sample (triplicates for each sample). Water served as a negative control in each run. After denaturation (95°C, 10 min), 50 cycles were performed (denaturation at 95°C, 15 s; primer annealing and extension at 60°C, 60 s; thermocycler CFX 384, Biorad, Munich, Germany). Fluorescence intensity was read out during each annealing/extension step. Target gene/β-actin expression ratios were determined by comparing C_*T*_ values of the target gene with those of the reference gene, β-actin. Relative quantification of different genes was determined according to the 2^ΔΔ*CT*^ method:

(1) XXtarget⁣/=β-⁢actinE/CTβ-⁢actinE,CTtarget

yielding a gene ratio with X being the input copy number and C_*T*_ the cycle number at threshold. The amplification efficiency for TARP γ2 was 1.89, for γ4 1.90, for γ7 1.92, for γ8 1.96, for CNIH-2 1.98, and for β-actin 1.94. For the AMPA receptor subunits the amplification efficiencies were 1.95 (GluA1, GluA3) and 1.96 (GluA2, GluA4).

### Data Analysis

The rectification index (RI) of rector currents was determined by comparing the chord conductance of agonist/blocker-sensitive currents at –70 and +70 mV or +40 mV according to:

(2a) RI=[I+70⁢m⁢V/(+70mV-E)rev]/[I/-70⁢m⁢V(-70mV-E)rev],

(2b) RI=[I+40⁢m⁢V/(+40mV-E)rev]/[I/-70⁢m⁢V(-70mV-E)rev],

where I is the amplitude of the currents at +70, +40, and −70 mV, respectively, and E_*rev*_ its extrapolated reversal potential.

Data were tested for Gaussian distribution by the Shapiro–Wilk tests and for homogeneity of variance with Levene’s test, followed by a two sample *t*-test or Mann–Whitney-*U*-test with or without Welch correction for equal or diverse variance. In case of paired data, a paired *t*-test or Wilcoxon signed rank test was performed. For group analyses, we used two-way ANOVA followed by Tukey or Holm’s *post hoc* test, in case of non-parametric data, aligned rank transformed ANOVA followed by Mann–Whitney-*U*-Test or Wilcoxon signed rank test with Holm’s correction for multiple comparisons. Tests were performed with the software R (R Development Core Team^1^). Non-Gaussian distributed data are displayed in box plots showing median (central line), quartiles (25 and 75%; box) and whiskers (±1.5 times the interquartile range). Statistical significance of gene expression by single-cell RT-PCR was analyzed by χ^2^ test. Significance level was set to *P* < 0.05. Except box plots, data are given as mean ± SD. n and N refer to the number of cells and mice, respectively.

## Results

### Pharmacological Characterization of Ca^2+^ Permeable AMPA Receptors in Brain Slices From Juvenile and Adult Mice

**FIGURE 1:**
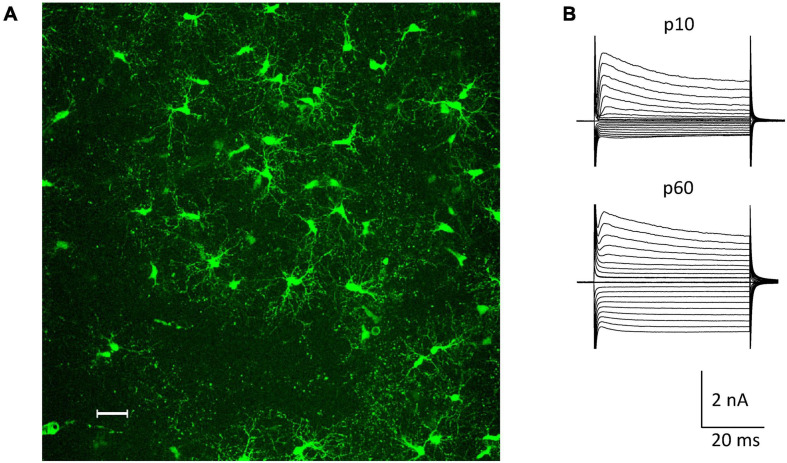
NG2 glia in the hippocampal CA1 region *in situ*. **(A)** Fluorescent NG2 glia in the stratum radiatum of a NG2ki-EYFP mouse at p78. Maximum intensity projection of 200 optical sections obtained by 2-photon-laser-scanning microscopy (950 nm excitation; Leica TSM, Leica Microsystems, Pulheim, Germany). Scale bar 30 μm. **(B)** Current patterns of NG2 glial cells obtained from the CA1 stratum radiatum of acute slices at p10 and p60 (de- and hyperpolarization from –160 to +20 mV; 10 mV increment, holding potential –70 mV).

The NG2ki-EYFP mouse line has been extensively characterized and allows for identification of NG2 glia prior to electrophysiological recordings and cell identification for flow cytometry (Karram et al., 2008). NG2^+^ cells located next to blood vessels were excluded from this study to avoid mix up with NG2^+^ pericytes (Figure 1A). NG2 glial cells were identified by their EYFP fluorescence, characteristic morphology and current pattern (small Na^+^ inward currents, prominent A-type K^+^ currents, inwardly rectifying K^+^ currents with the latter increasing with age; e.g. (Moshrefi-Ravasdjani et al., 2017; Seifert and Steinhäuser, 2018; Figure 1B).

**FIGURE 2:**
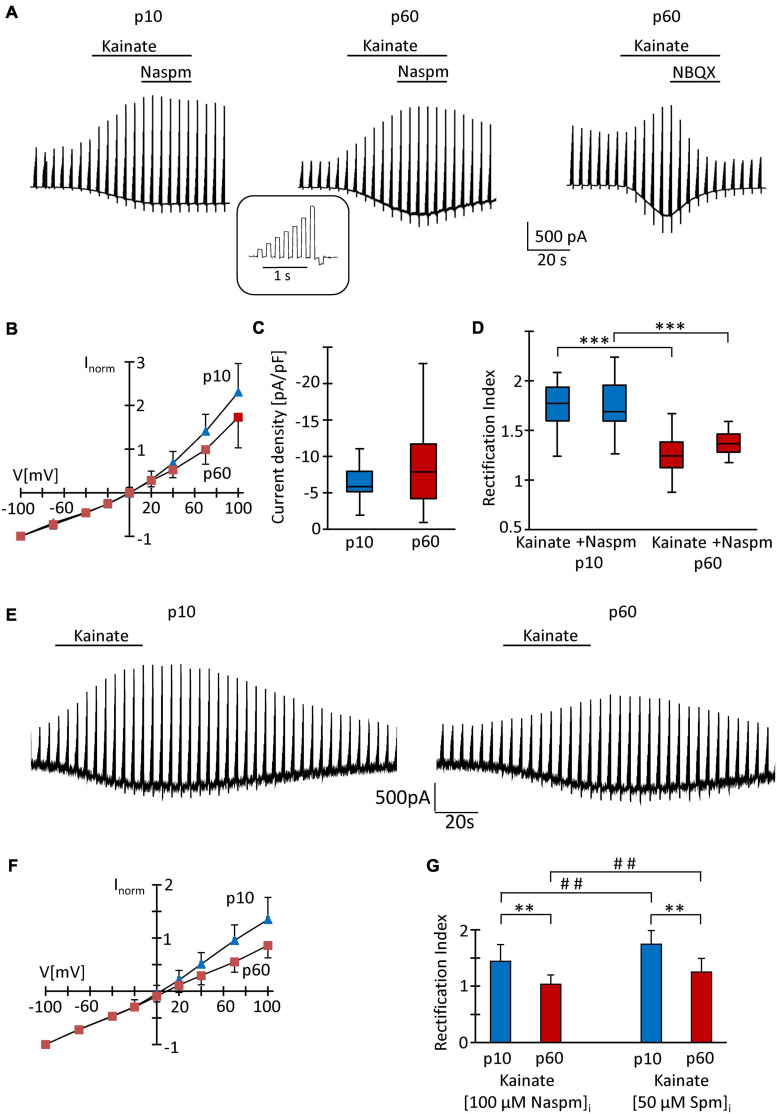
Analysis of AMPA receptors in hippocampal NG2 glia *in situ*. **(A)** After incubation in blockers of K^+^- and Na^+^-channels (200 μM quinine, 100 μM Ba^2+^, 0.5 μM TTX) and GABA_*A*_ receptors (100 μM picrotoxin), kainate (250 μM) and Naspm (100 μM) were applied to slices as indicated while the membrane of the patched cell was de- and hyperpolarized (–70, –40, –20, 0, 20, 40, 70 100, and –100 mV; duration 100 ms, separated by 100 ms, every 4.5 s; see inset). The pipette solution contained spermine (50 μM). Representative responses are shown from cells at p10 (left) and p60 (middle, right). NBQX (10 μM) almost completely (by 97%) blocked kainate-induced currents (right). **(B)** To obtain I/V relations of kainate-induced responses, currents before and during agonist application were subtracted at corresponding voltages. Responses were normalized to maximum inward currents and averaged (p10, blue, *n* = 20; p60, red, *n* = 41). **(C)** Current density of AMPA receptor currents at –70 mV did not differ between p10 (–5.85 pA/pF, *n* = 20) and p60 (–7.86 pA/pF, *n* = 41). **(D)** Rectification of the I/V curves significantly differed between p10 (kainate: RI = 1.77; kainate + Naspm: RI = 1.69, *n* = 20) and p60 (kainate: RI = 1.24, *n* = 41; kainate + Naspm: RI = 1.37, *n* = 8). Within individual age groups, application of Naspm did not affect RI. Significant differences are indicated by asterisks (*p* < 0.001). **(E)** Same conditions as described in panel **(A)**, but with Naspm (100 μM) instead of spermine in the pipette solution. **(F)** Kainate responses were determined as described in panel **(B)** (p10, blue, *n* = 18; p 60, red, *n* = 18). **(G)** Rectification of I/V curves at p10 (RI = 1.44, *n* = 18) and p60 (RI = 1.03, *n* = 18). Significant differences are indicated [between ages: ^∗∗^*p* < 0.01, ^∗∗∗^*p* < 0.001; between (Naspm)_*i*_ and (spermine)_*i*_: ## *p* < 0.01].

At p10, adding the K^+^ channel blockers quinine (200 μM) and Ba^2+^ (100 μM) to the bath shifted the resting potential of the NG2 glial cells from −85 mV (median; quartiles: −75–−90 mV) to −50 mV (−55 to −44 mV) and increased the input resistance from 225 MΩ (165–320 MΩ) to 1,000 MΩ (833–2,000 MΩ, *n* = 26 cells, *N* = 6 mice). Subsequent application of kainate (250 μM) induced inward currents of −5.85 pA/pF (−5.14 to −7.93 pA/pF) (*V* = −70 mV; *n* = 20, *N* = 6) (Figures 2A,C). Although spermine was added to the pipette solution (50 μM) to block outward currents through Ca^2+^ permeable receptors, we still observed significant outward rectification (RI = 1.77, 1.60–1.94) (Figures 2B,D). The reversal potential of the responses was −0.1 mV (−1.2 to 7.0 mV; Figure 2B). Increasing the spermine concentration to 100 μM did not change rectification (1.58, 1.44–1.86, *n* = 6, *N* = 2, *p* = 0.298). Next we co-applied kainate (250 μM) with 1-naphtyl-acetyl-spermine (Naspm; 100 μM) to detect putative currents through Ca^2+^ permeable AMPA receptors (Koike et al., 1997). While the reversal potential slightly increased in the presence of Naspm (from −0.1 mV, −1.2–7.0 mV to 4.8 mV, 0.1–12.8 mV; *n* = 20, *p* < 0.001), RI did not change (1.69, 1.59–1.96, *n* = 20, *N* = 5) (Figure 2D). Naspm reduced the kainate-induced currents by 8.1 ± 12.1% (*V* = −100 mV; Figure 2A, left).

At p60, the K^+^ channel blockers induced a depolarization from −87 mV (−84 to −90 mV) to −65 mV (−70 to −52 mV) while the input resistance increased from 80 MΩ (66–104 MΩ) to 714 MΩ (625–1,000 MΩ, *n* = 41, *N* = 9; *p* < 0.001). The lower input resistance in these older mice reflected the cells’ increased Kir conductance (Seifert and Steinhäuser, 2018). Application of kainate (250 μM, with 50 μM spermine in the pipette) induced inward currents of −7.86 pA/pF (−70 mV; −4.20 to −11.7 pA/pF), which reversed current direction at −0.2 mV (−3.2–7.3 mV), showed an RI of 1.24 (1.12–1.38, *n* = 41, *N* = 9) und were completely blocked by NBQX (10 μM) (Figures 2A–D). This stronger rectification compared to the juvenile stage (*p* < 0.001) might have indicated a more efficient spermine block and hence an elevated Ca^2+^ permeability in NG2 glia from the older mice. In the presence of Naspm, RI was 1.36 (1.28–1.46, *n* = 8; *N* = 3), i.e., lower than at juvenile stage (Figure 2D), but the efficiency of Naspm-dependent inhibition of kainate-induced currents (by 15.5 ± 16.8% at *V* = −100 mV) did not change during maturation.

Recently it was reported that intracellular application of Naspm completely blocks outward currents through GluA2-lacking, Ca^2+^ permeable AMPA receptors and that this block, in contrast to intracellular spermine block, does not depend on TARP expression (Coombs et al., 2021). Indeed, with Naspm (100 μM) in the pipette solution (Figures 2E,F), we observed stronger rectification of kainate-induced responses (p10: RI = 1.44 ± 0.30, *n* = 18, *N* = 3, *p* < 0.01; p60: RI = 1.03 ± 0.17, *n* = 18, *N* = 3, *p* < 0.01). At p60, the blocking effect of intracellular Naspm was also stronger than at p10 (*p* < 0.01) (Figures 2F,G).

Intracellular polyamines block outward currents through Ca^2+^ permeable, GluA2-lacking AMPA receptors (Bowie and Mayer, 1995; Donevan and Rogawski, 1995; Kamboj et al., 1995), which reduces rectification (Koh et al., 1995; Toth and McBain, 1998; Soto et al., 2007). Thus, in line with previous findings (Seifert and Steinhäuser, 1995; Seifert et al., 2003), our data confirm a low Ca^2+^ permeability of AMPA receptors in juvenile NG2 glia which, judged by the lower RI, increased by adulthood.

### Developmental Regulation of TARPs and CNIHs in Hippocampal NG2 Glia

**FIGURE 3:**
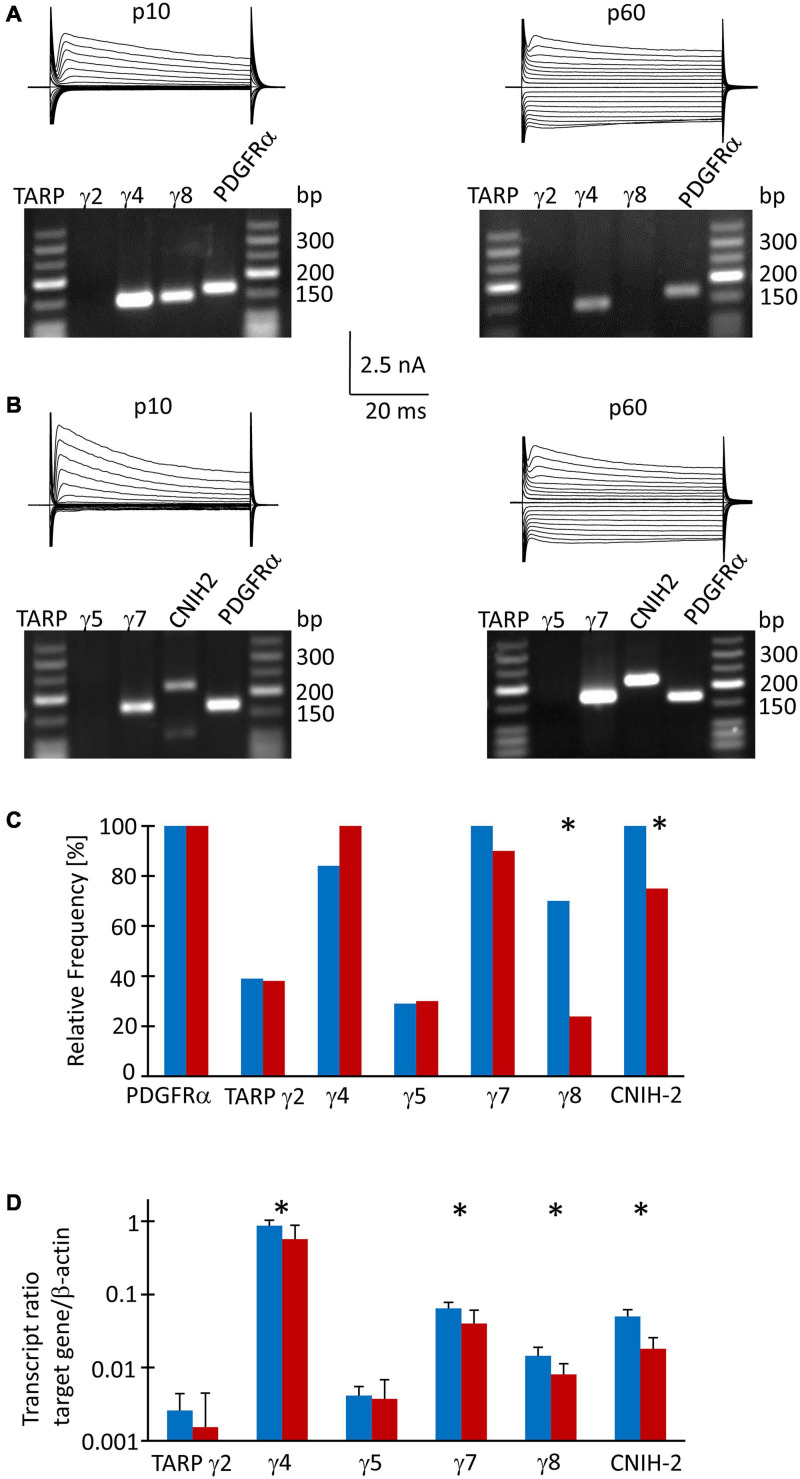
TARP/CNIH expression in individual und FAC sorted NG2 glia. **(A,B)** Membrane currents were evoked through de- and hyperpolarization of the cell membrane (–160 to 20 mV, 10 mV increments, duration 50 ms, holding potential –70 mV). After recording *in situ*, the cell content was harvested and RT-PCR was performed. The agarose gels show the respective transcript patterns. PDGFα receptor mRNA served as a positive control. The PCR products matched the predicted lengths (Supplementary Table 1). **(C)** Relative frequencies of TARP- and CNIH-2 expression by individual cells (p10: γ2, *n* = 38 cells; γ4 and γ8, *n* = 43; γ5 and γ7, *n* = 24; CNIH-2, *n* = 19. p60: γ2, γ4 and γ8, *n* = 20; γ5, γ7 and CNIH-2, *n* = 21). **(D)** qPCR was performed with FAC sorted NG2 glial cells. Expression ratios were determined according to equation (1). Mean and SD were determined at p10 (*n* = 8) and p60 (*n* = 12). Significant differences are indicated by asterisks (*p* < 0.05).

Since TARPs and CNIHs affect the intracellular polyamine block and may accompany Ca^2+^ permeable AMPA receptor subunits, we analyzed their expression by NG2 glial cells. First, multiplex single cell RT-PCR was performed after electrophysiological characterization of the cells in acute slices, using PDGFRα mRNA as a NG2 glia-specific marker. This marker was found in all cells harvested (p10, *n* = 64; p60, *n* = 43). We found heterogeneous expression of auxiliary subunits in NG2 glia. At p10, a majority of cells expressed TARP γ4 (80%, *n* = 43), TARP γ7 (100%, *n* = 24), TARP γ8 (67%, *n* = 43), and CNIH-2 (100%, *n* = 19) while TARP γ2 and TARP γ5 were less frequent (37%, *n* = 38 and 29%, *n* = 24, respectively) (Figures 3A,B). At p60, a similar expression pattern was observed: TARP γ4 (95%, *n* = 21), TARP γ7 (86%, *n* = 20) and CNIH-2 (71%, *n* = 20) were most abundant while TARPs γ2 and γ5 were less frequent (36%, *n* = 21 and 29%, *n* = 20). However, the expression of TARP γ8 differed, declining to 23% at p60 (*n* = 21, *p* = 0.0005), and CNIH-2 was also less abundant than at p10 (*n* = 20, *p* = 0.02) (Figure 3C).

To receive quantitative information as to the expression of the auxiliary subunits, real-time PCR was performed with cDNA from FAC sorted NG2 glial cells isolated from the hippocampus of EYFP mice, using β-actin as a housekeeping gene. At p10, TARP γ4 was most abundant (expression ratio 0.864 ± 0.166, *n* = 8), followed by TARP γ7 (0.064 ± 0.014, *n* = 8) and CNIH-2 (0.05 ± 0.011, *n* = 8). Expression of the hippocampus-specific subunit γ8 was low in NG2 glia (0.014 ± 0.004, *n* = 8). TARP γ2 and γ5, which are abundantly present in the cerebellum, were expressed at a low level while TARP γ3 was absent (Figure 3D). At p60, the relation between the individual auxiliary subunits remained similar, although expression was generally lower (TARP γ4, 0.570 ± 0.313, *n* = 12; TARP γ7, 0.040 ± 0.021, *n* = 12; CNIH-2, 0.018 ± 0.008, *n* = 12; TARP γ8, 0.008 ± 0.003, *n* = 12) (Figure 3D). Expression of TARP γ2 and TARP γ5 was close to the detection threshold and showed no developmental regulation.

Next, we compared expression of mRNA for the subunits GluA1-4 in FAC-sorted NG2 glia from mice at both ages, using again β-actin as a housekeeping gene. At p10, GluA2 was most abundant (expression ratio 0.102 ± 0.059), followed by GluA3 (0.050 ± 0.023), GluA4 (0.030 ± 0.014) and GluA1 (0.017 ± 0.008, *n* = 10 for all) (Supplementary Figure 1). A similar expression pattern was found at p60 (GluA2, 0.098 ± 0.066; GluA3, 0.035 ± 0.025; GluA4, 0.025 ± 0.018; GluA1, 0.013 ± 0.018; *n* = 10 for all) (Supplementary Figure 1).

### Pharmacological Characterization of Ca^2+^ Permeable AMPA Receptors in NG2 Glia Freshly Isolated From p10 Mice

Because the slow application of the receptor blockers, diffusion barriers and putative indirect effects of drug application in slices might have compromised the results, we characterized properties of AMPA receptors also in acutely isolated NG2 glial cells. The developmental differences in TARP γ8 expression also led us to test the effect of a TARP γ8 antagonist, JNJ55511118 (further on called JNJ; Maher et al., 2016), in addition to the more global blockers of Ca^2+^ permeable AMPA receptors, Naspm and IEM-1460 (Magazanik et al., 1997).

**FIGURE 4:**
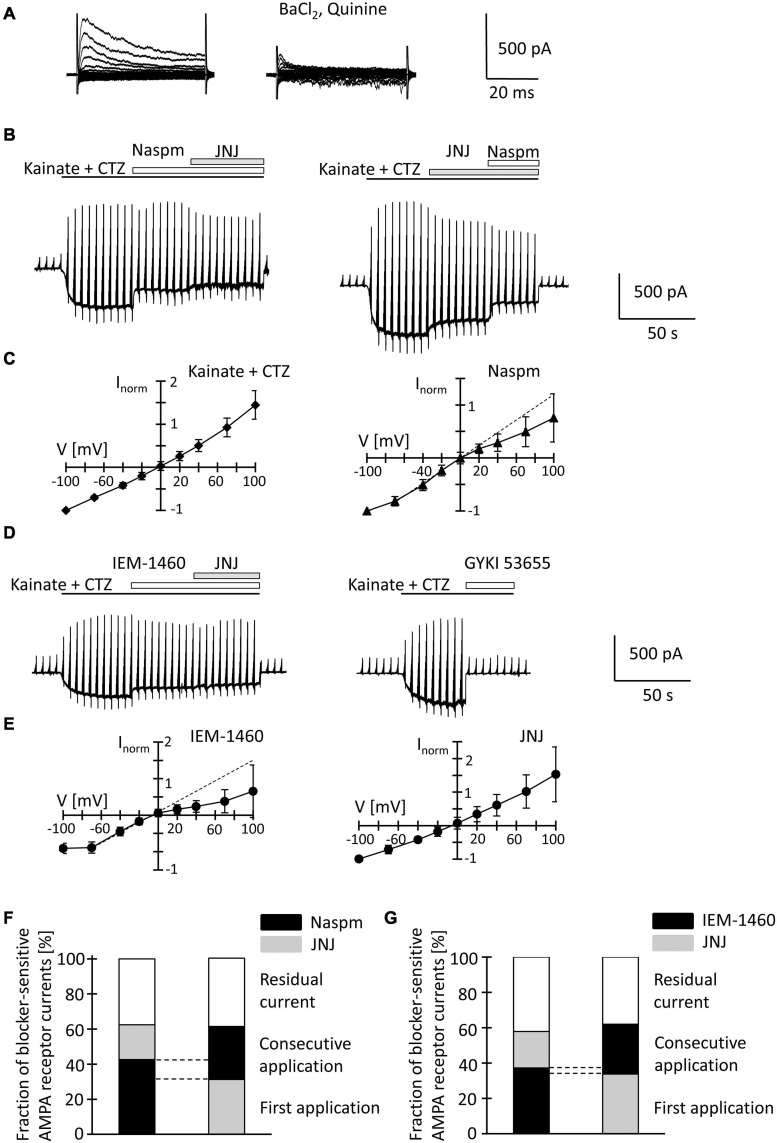
Inhibition of AMPA receptor currents by blockers of Ca^2+^ permeable receptors and a TARP γ8 inhibitor in freshly isolated NG2 glia at p10. **(A)** Membrane currents were elicited as described in Figure 2 (left, normal bath solution; right, after adding 100 μM quinine and 10 μM BaCl_2_). **(B)** The membrane was de- and hyperpolarized as explained in Figure 2A and kainate (250 μM), CTZ (100 μM), Naspm (50 μM) and the TARP γ8 inhibitor, JNJ 55511118 (10 μM), were applied as indicated. **(C)** shows mean I/V relations of kainate-induced current (*n* = 35; left) and the Naspm-sensitive component (*n* = 21; right). Currents were normalized to maximal inward currents in each cell. The blocker-sensitive currents were determined by successively calculating the difference between the current amplitude in the presence of the blocker and the amplitude in the presence of the preceding substance. **(D)** Using the protocols explained in panel **(B)**, AMPA receptor currents were elicited and blocked by adding IEM-1460 (100 μM), JNJ (10 μM), and GYKI 53655 (100 μM). **(E)** Mean I/V relations of IEM-1460- (*n* = 17; left) and JNJ-sensitive currents (*n* = 19; right). **(F,G)** Relative fractions of AMPA receptor currents sensitive to Naspm, IEM-1460 and JNJ. The order of consecutive applications has been reversed. Overlapping sensitivity to both blockers is indicated by dashed lines. The relative respective inhibition was averaged [**(F)** *n* = 6 and 7; **(G)** *n* = 10 and 6, respectively]. Only those cells are shown in which both blockers could be successfully applied consecutively.

After fresh isolation, NG2 glia was identified by their intrinsic fluorescence. At p10, addition of quinine and Ba^2+^ (100 μM both) to the bath depolarized the cells from −28.2 ± 15.8 mV to −16.2 ± 8.0 mV and increased their input resistance from 1,285 ± 847 MΩ to 2,152 ± 2,044 MΩ (*n* = 35, *N* = 13) (Figure 4A). With spermine (50 μM) in the pipette, kainate (250 μM) together with the AMPA receptor modulator cyclothiazide (100 μM) induced inward currents of 161.4 ± 124.4 pA/pF (*n* = 35, *N* = 13, *V* = −70 mV) (Figure 4B). The I/V relations of the kainate-induced responses revealed a reversal potential (V_*rev*_) of −1.5 ± 8.9 mV and RI = 1.26 ± 0.27 (*n* = 35, *N* = 13), confirming previous findings (Figure 4C; Seifert et al., 2003). GYKI53655 reduced the AMPA receptor currents by 97 ± 4% (*n* = 11, *N* = 9) (Figure 4D), with the RI of GYKI-sensitive currents being 1.44 ± 0.46 (*V*_*rev*_ = −2.8 ± 10.2 mV).

To further characterize expression of Ca^2+^ permeable AMPA receptors and putative involvement of TARP γ8 in kainate-induced responses of NG2 glia, JNJ was applied subsequent to Naspm or IEM-1460. Naspm (50 μM) decreased the inward currents (by 53.1 ± 18.3%, *V* = −70 mV, *n* = 15, *N* = 11) while the subsequent application of JNJ (10 μM) led to a further block of the kainate-induced currents (by 19.8 ± 14.7%, *n* = 6, *N* = 5) (Figure 4B). With the blockers in reverse order, JNJ at 10 μM reduced currents by 30.5 ± 6.9% (*n* = 8, *N* = 5) while addition of Naspm led to a further reduction by 30.2 ± 11.8% (*n* = 7; *N* = 4) (Figure 4B). To avoid putative non-specific effects, we reduced the JNJ concentration to 1 μM. Under these conditions, JNJ reduced the currents by 15.1 ± 5.3% (*n* = 10, *N* = 4, *V* = −70 mV), which was less compared to block by 10 μ JNJ (*p* < 0.001; Supplementary Figure 2C). The Naspm (50 μM)-mediated block of JNJ (1 μM)-insensitive currents was not different from the values obtained with 10 μM JNJ (36.8 ± 11.4%, *n* = 9; *N* = 4) (Supplementary Figures 2A,C). These data demonstrated that despite the inefficacy of intracellular spermine, extracellularly applied spermine analogs profoundly affected the receptor currents in juvenile hippocampal NG2 glia. Naspm and JNJ showed an additive inhibition. The reversal potential of the Naspm-sensitive responses was 0.2 ± 8.9 mV, with RI = 0.55 ± 0.27 mV (*n* = 21, *N* = 11) (Figure 4C), indicating that these currents were indeed mediated by Ca^2+^ permeable receptors. The corresponding I/V relations of JNJ-sensitive currents is outward rectifying (*V*_*rev*_ = −7.2 ± 14.1 mV, RI = 1.28 ± 0.36, *n* = 12, *N* = 6) (Figure 4E). Notably, a significant proportion of the responses remained in the presence of both blockers (10 μM JNJ: 38.3 ± 19.2%, *n* = 13, *N* = 6; 1 μM JNJ: 45.9 ± 11.1%, *n* = 11, *N* = 4) (Figures 4F,G; Supplementary Figure 2C).

To confirm the above findings another, structurally related blocker of Ca^2+^ permeable AMPA receptors was used, IEM-1460. IEM-1460 (100 μM) blocked 38.8 ± 10.8% of the kainate responses (*V* = −70 mV, *n* = 11, *N* = 4). Compared to Naspm, this blocking efficacy was lower (*p* = 0.03). Subsequent application of JNJ further reduced the receptor currents (by 20.6 ± 9.2%, *n* = 10, *N* = 4) (Figure 4D). Vice versa, if JNJ was applied first, the responses were reduced by 34.7 ± 6.7% (*n* = 7; *N* = 4) and additionally by 28.2 ± 10% through IEM-1460 (*n* = 6, *N* = 4). Again, 40.5 ± 9.4% (*n* = 16, *N* = 4) of the responses were insensitive to both inhibitors (Figure 4G). IEM-sensitive currents reversed at −3.9 ± 6.1 mV and showed inward rectification (RI = 0.4 ± 0.32, *n* = 17, *N* = 4) (Figure 4E). In contrast, JNJ-sensitive currents were again outward rectifying (RI = 1.31 ± 0.55, *V*_*rev*_ = −3.9 ± 8.2, *n* = 16, *N* = 4) (Figure 4E).

JNJ still blocked part of the Naspm- or IEM-1460-insensitive currents. Vice versa, after initial application of JNJ, Naspm and IEM-1460 still blocked Ca^2+^ permeable receptors. Thus, TARP γ8 is mainly associated with Ca^2+^- impermeable AMPA receptors.

### Pharmacological Characterization of Ca^2+^ Permeable AMPA Receptors in NG2 Glia Freshly Isolated From Mice at p60

**FIGURE 5:**
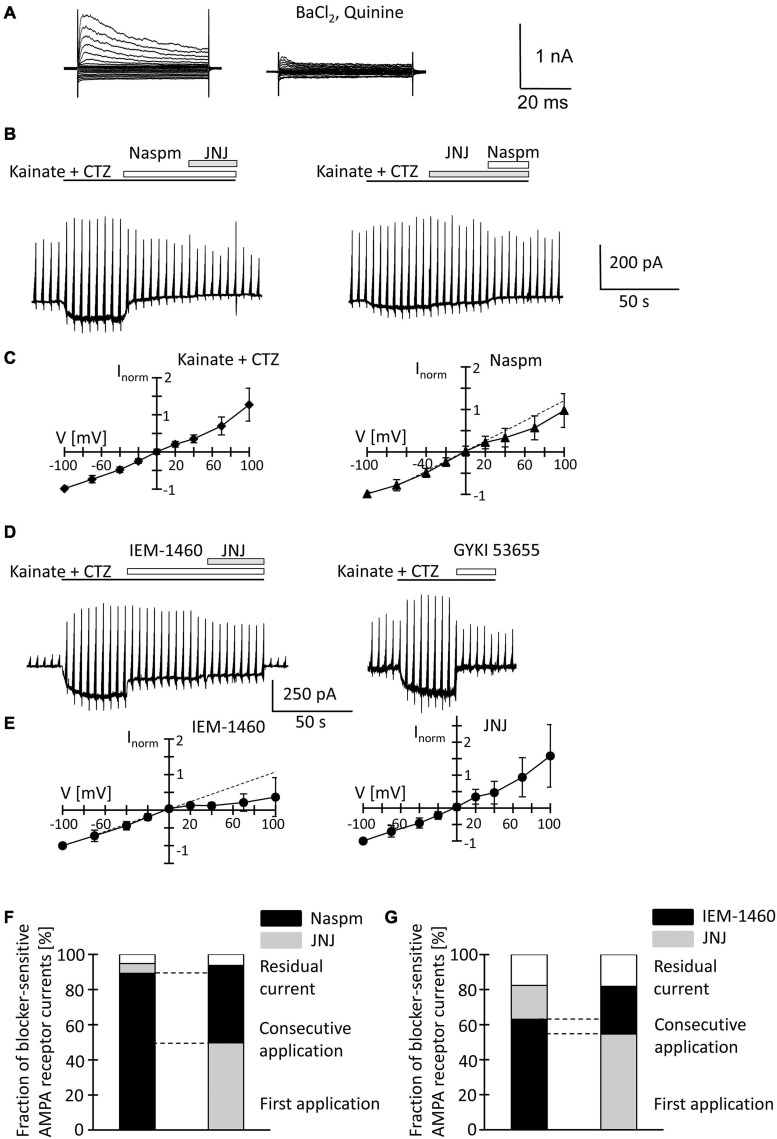
Inhibition of AMPA receptor currents by blockers of Ca^2+^ permeable receptors and a TARP γ8 inhibitor in freshly isolated NG2 glia at p60. **(A,B,D)** Stimulation and drug application were as described for p10 (Figure 4). **(C,E)** I/V relations for responses evoked by kainate + CTZ (*n* = 20, left), Naspm- (*n* = 15), IEM-1460- (*n* = 12), and JNJ-sensitive currents (*n* = 8). **(F,G)** Relative current inhibition was only compared for those cells in which both blockers could be successfully applied consecutively [**(F)** *n* = 4 and 5; **(G)** *n* = 6 and 6].

At p60, quinine and Ba^2+^ depolarized the cells (from −23.6 ± 11.5 mV to −15.9 ± 8.1 mV) and increased the input resistance (from 877 ± 418 MΩ to 1,958 ± 1,416 MΩ; *n* = 20, *N* = 10) (Figure 5A). Co-application of kainate and cyclothiazide induced similar inward currents as at p10 (191.1 ± 182.8 pA/pF, *V* = −70 mV, *n* = 20, *N* = 10). With spermine (50 μM) in the pipette solution, the kainate-induced currents reversed at −0.5 ± 6.0 mV. RI was 0.98 ± 0.26 (*n* = 20) indicating linear I/V relationships, which differed from the outward rectifying responses seen in juvenile NG2 glia (Figures 5B,C; *p* = 0.0005). GYKI53655 almost completely blocked the responses (by 97 ± 5%, *n* = 6, *N* = 4). RI of GYKI-sensitive currents was 1.00 ± 0.13 (*V*_*rev*_ = −2.8 ± 8.3 mV) (Figure 5D).

Naspm (50 μM) exerted a much stronger inhibition than at p10 (by 77.4 ± 15.2%, −70 mV, *n* = 10, *N* = 6, *p* = 0.002). Subsequent co-application of JNJ (10 μM) led to further reduction of the responses (by 5.6 ± 5.7%, *n* = 4, *N* = 3) (Figure 5B). With the blockers applied in reverse order, 10 μM JNJ reduced currents by 50.5 ± 20.0% (*n* = 6, *N* = 3) while subsequent co-application of Naspm further reduced the currents by 44.0 ± 24.0% (*n* = 5, *N* = 3) (Figure 5B). At a concentration of 1 μM, JNJ-induced inhibition (by 24.4 ± 8.7%; *n* = 10, *N* = 4) was weaker (*p* < 0.05) (Supplementary Figures 2B,C), but more efficient than at p10 (*p* < 0.05). Subsequent block by Naspm (50 μM) was as efficient as after application of JNJ at the higher concentration (by 53.8 ± 9.7%, *n* = 9, *N* = 4), but stronger than at p10 (*p* = 0.01). The reversal potential of Naspm-sensitive currents was −1.3 ± 9.0 mV, with RI = 0.74 ± 0.39 mV (*n* = 15; *N* = 7), indicating that the current were partially mediated by Ca^2+^ permeable receptors. JNJ-blocked currents reversed at −1.4 ± 7.1 mV and showed outward rectification (RI = 1.20 ± 0.51, *n* = 8; *N* = 3) (Figures 5C,D). The proportion of blocker-insensitive receptor currents was much smaller than at p10 (5.8 ± 10.1%, *n* = 9, *N* = 3, *p* = 0.0001) (Figure 5F). In 6/9 cells, inhibition of the responses was even virtually complete. These results indicate that AMPA receptors in NG2 glia at p60 show a higher Ca^2+^ permeability, and that JNJ may also inhibit part of the Naspm-sensitive currents.

IEM-1460 (100 μM) inhibited 63.1 ± 6.4% (*V* = −70 mV, *n* = 6, *N* = 3) of the receptor currents, thus being more efficient than at p10 (*p* < 0.001) but less efficient than Naspm at p60 (*p* = 0.028). IEM-sensitive currents showed strong inward rectification (RI = 0.30 ± 0.31, *V*_*rev*_ = −2.9 ± 7.6 mV, *n* = 12, *N* = 3) (Figures 5D,E). Subsequent co-application of JNJ exerted additional inhibition (by 19.3 ± 7.3%) and the JNJ-sensitive currents were again outwardly rectifying (RI = 1.46 ± 0.91, *V*_*rev*_ = −1.3 ± 7.1, *n* = 12) (Figures 5D,E). When JNJ was applied before adding IEM-1460, inhibition was 54.7 ± 5.4 and 27.2 ± 6.1% (*n* = 6, *N* = 3). Inhibition by JNJ was more effective than at cells at p10 (*p* = 0.00007), and the proportion of currents insensitive to the co-applied blockers was lower than at the early stage (10 μM JNJ: 17.8 ± 8.2%, *n* = 12, *N* = 3, *p* < 0.001; 1 μM JNJ: 21.8 ± 8.0%, *n* = 12, *N* = 4, *p* < 0.001) (Figure 5G and Supplementary Figure 2C). In contrast to Naspm, consecutive application of IEM-1460 and JNJ showed only a small overlap (Figures 5D,G).

Together, the data demonstrate that extracellularly applied spermine analogs inhibit AMPA receptors in hippocampal NG2 glia. The efficiency of inhibition by Naspm and IEM-1460 was higher in NG2 glia from adult mice. Naspm and JNJ showed a partly overlapping block. Since there was almost no overlap of IEM-1460- and JNJ-mediated inhibition, it seems that Naspm also blocked some Ca^2+^ impermeable receptors. In line with the observations at p10, the data suggested that JNJ blocked Ca^2+^ impermeable receptors. Although molecular analysis revealed developmental downregulation of TARP γ8, the modulatory effect of JNJ was stronger in adult NG2 glia.

### Ca^2+^ Permeability of Glial AMPA Receptors

**FIGURE 6:**
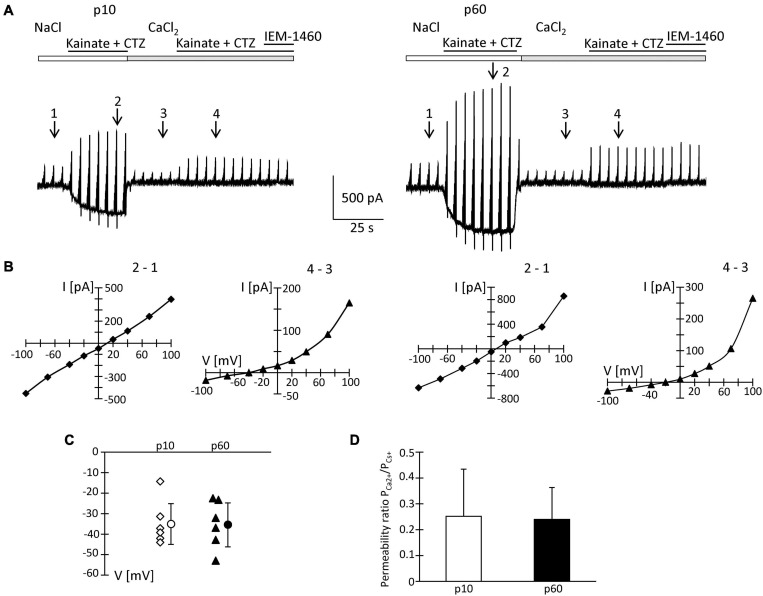
Ca^2+^ permeability of kainate-activated membrane currents. **(A)** Receptor currents were elicited by co-applying kainate (250 μM) and CTZ (100 μM) to NG2 glia freshly isolated at p10 and p60 (cf. Figures 2, 4 for stimulation protocols). After initial recording in standard bath solution, high CaCl_2_ (50 mM) solution was applied. At holding potential, kainate/CTZ induced inward currents of 325 and 500 pA (standard solution) or 15 and 20 pA (high Ca^2+^ solution) at p10 and p60, respectively. **(B)** I/V relations of the cells shown in panel **(A)** were determined by subtracting the current amplitudes at corresponding voltages at the indicated time points (in standard solution: 2–1; in Ca^2+^ solution: 4–3). **(C)** Reversal potentials (V) of receptor currents obtained in high CaCl_2_ solution were plotted as a function of postnatal age. Bars indicate mean ± SD (*n* = 6 for each age group). **(D)** Bar graph give the ratio P_*Ca2*__+_/P_*Cs*__+_ with mean ± SD of AMPA receptor currents elicited in high Ca^2+^ solution. In these experiments, intracellular K^+^ was replaced by Cs^+^ and TEA.

To quantify the Ca^2+^ permeability of NG2 glia AMPA receptors, Na^+^-free extracellular solution with Ca^2+^ (50 mM) as the sole charge carrier was used. Kainate/CTZ induced currents in high Ca^2+^ solution, indicating Ca^2+^ permeability of the glial AMPA receptors. The permeability coefficient P_*Ca*_/P_*Cs*_ was calculated according to the constant field equation (Mayer and Westbrook, 1987; Seifert and Steinhäuser, 1995). In juvenile mice, the ratio I_*Ca*_/I_*Na*_ of kainate-evoked responses was 4.1 ± 1.2% (Figure 6A). The I/V relations changed from a linear shape in Na^+^ solution to strong outward rectification in high Ca^2+^ solution (Figure 6B). The reversal potential of kainate/CTZ-evoked responses in high Ca^2+^ solution was −34.7 ± 10.0 mV, and the permeability ratio P_*Ca*_/P_*Cs*_ amounted to 0.25 ± 0.18 (*n* = 6; *N* = 4) (Figures 6C,D).

Since the inhibitory effects of Naspm and IEM-1460 were stronger at p60, we performed the same analysis at the older stage. Here, we found I_*Ca*_/I_*Na*_ = 5.5 ± 1.6%, a reversal potential in high Ca^2+^ solution at −35.1 ± 10.7 mV, and P_*Ca*_/P_*Cs*_ = 0.24 ± 0.13 (*n* = 6; *N* = 5) (Figures 6A,C,D). None of those parameters differed from the juvenile stage and they were in accordance with previous data (Seifert et al., 1997b). Thus, the overall Ca^2+^ permeability of the receptors was similar at both stages. However, in NG2 glia from older mice, the higher blocking efficacy of Naspm and IEM-1460 and the enhanced polyamine block indicated expression of a mosaic of Ca^2+^ permeable and impermeable receptors.

### Characterization of Postsynaptic Responses in NG2 Glia of the Hippocampal CA1 Region

**FIGURE 7:**
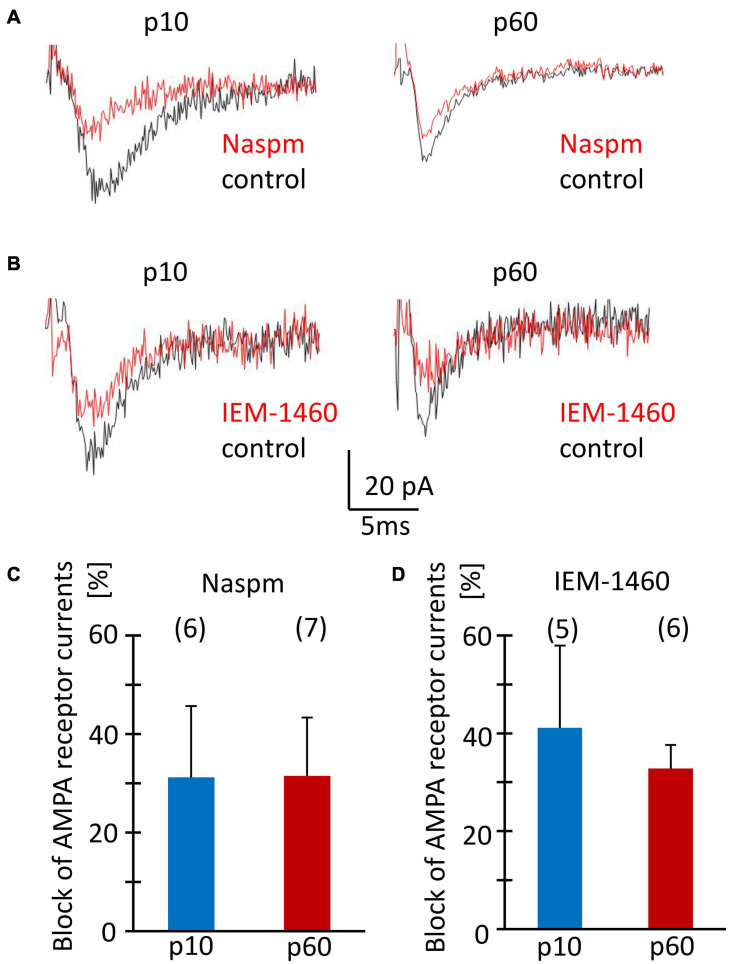
Sensitivity to Naspm and IEM-1460 of synaptic AMPA receptor currents in NG2 glia *in situ*. **(A,B)** Stimulation of Schaffer collaterals in the stratum radiatum of the CA1 region elicited ePSCs in NG2 glial cells (holding potential –70 mV). Bath solution contained picrotoxin (100 μM) to inhibit GABA_*A*_ receptors, and CTZ (100 μM) to increase AMPA receptor responses. Bath application of Naspm (50 μM) or IEM-1460 (100 μM) reduced ePSCs. **(C,D)** Mean and SD of peak ePSC inhibition by Naspm and IEM-1460 in NG2 glia of slices from mice at p10 and p60. Number of cells is given in parentheses. The pipette solution contained CsCl to block K^+^ channels. The sensitivity to Naspm and IEM-1460 did not change during development (two-tailed *t*-test).

Acute isolation of cells from the tissue shears off many of the fine processes. Thus, the properties reported above should have largely represented receptors located close to the soma, while previous work found them mostly at postsynapses on the processes (Haberlandt et al., 2011). Therefore, we tested the sensitivity of postsynaptic currents of NG2 glia to Naspm and IEM-1460. Stimulation of Schaffer collaterals was performed in the presence of cyclothiazide (100 μM) and picrotoxin (100 μM). Naspm (50 μM) and IEM-1460 (100 μM) similarly reduced synaptic currents (p10, by 31.2 ± 14.4%, *n* = 6, *N* = 4 and 41.1 ± 16.8%, *n* = 5, *N* = 3; p60, by 31.5 ± 10.8%, *n* = 7, *N* = 4 and 32.8 ± 4.8%, *n* = 6, *N* = 5) (Figures 7A–D). The efficiency of inhibition was similar for IEM-1460 and Naspm and for both developmental stages. Thus, after minimal stimulation at a frequency 0.1 Hz, both blockers of Ca^2+^ permeable receptors inhibited evoked synaptic AMPA receptor responses.

## Discussion

Previous functional analyses showed that early after birth, NG2 glia express Ca^2+^ permeable AMPA receptors (Seifert et al., 2003), although further details, particularly the expression of auxiliary subunits were not known. Here we addressed the question whether NG2 glial cells in the hippocampus express TARP and CNIH subunits and whether expression changes during postnatal maturation. The data demonstrate abundant expression of TARPs γ4, γ7, γ8, and CNIH-2, which declined by maturity. Functional analyses using spermine analogs suggested enhanced expression of Ca^2+^ permeable receptors in NG2 glia of the adult hippocampus.

### TARP and CNIH Expression in Glial Cells

The first auxiliary transmembrane protein described to interact with AMPA receptors was stargazin, later termed TARP γ2, which is responsible for surface expression of AMPA receptors in cerebellar granule cells (Chen et al., 2000). TARP γ2 has a PDZ-binding site, which interacts with PSD-95 and also accounts for synaptic clustering (Schnell et al., 2002). While TARP γ2 is abundantly expressed by cerebellar granule and Purkinje cells, it is less present in other brain regions. Meanwhile, a whole gene family of TARPs was identified (Tomita et al., 2003). Because NG2 glia receives synaptic input from neurons through their AMPA receptors the question of glial TARP expression emerged. TARP γ4 was found in glial cells of cerebellum, hippocampus, and neocortex (Tomita et al., 2003; Fukaya et al., 2005). Our molecular analysis extended these findings by revealing that in the hippocampus, NG2 glia express TARPs, with TARP γ4 being most abundant, followed by TARPs γ7, γ8, and CNIH-2. Predominant expression of TARPs γ4 and γ7 was also found with RNA-Seq analysis (Larson et al., 2016). A characteristic feature of γ4 is its strong expression in the embryonic and early postnatal brain and subsequent down-regulation. Expression of CNIH-2 similarly decreased during development, both on the single cell level and in FAC-sorted NG2 glia.

### Consequences of TARP/CNIH Expression for AMPA Receptor Function

In our previous work we have reported molecular and functional properties of AMPA receptors in NG2 glia of the juvenile hippocampus, including Ca^2+^ permeability, splicing, and desensitization kinetics (Seifert and Steinhäuser, 1995; Seifert et al., 1997a, b; Matthias et al., 2003). Some of the earlier data appeared contradictory, e.g., the finding that enhanced expression of GluA2 flip did not entail changes in receptor desensitization (Seifert et al., 2003). The heterogeneous expression of auxiliary subunits identified in the present study might explain this apparent discrepancy, because they profoundly affect receptor function.

In heterologous expression systems, co-expression of TARP γ4 with GluA2 conferred high kainate efficacy to the receptor complex, slowed its desensitization and favored incomplete desensitization while rectification of the I/V relations was not altered (Korber et al., 2007). TARP γ7 is highly expressed in the cerebellum where it binds to the scaffold protein PSD-95 and supports synaptic targeting of AMPA receptors (Kato et al., 2007). It is tempting to speculate that TARP γ7 has a similar role in NG2 glia where it is abundantly expressed according to our data. TARP γ8 is highly expressed in the hippocampus, both at asymmetrical postsynaptic and extrasynaptic sites. Deletion of this subunit reduced AMPA receptor density, synaptic localization and impaired the induction of long term potentiation (LTP) (Rouach et al., 2005; Fukaya et al., 2006). Both TARP γ7 and γ8 interact with AMPA receptors to increase their CTZ potentiation and kainate efficacy (Gill et al., 2011). TARP γ7 modulates GluA2 containing receptors in a subunit composition specific manner (Kato et al., 2008). In contrast to hippocampal neurons, expression of TARP γ8 in NG2 glia was lower than TARP γ4 and γ7, and strongly downregulated until adulthood.

While CNIH-2 is expressed in astrocytes of the hippocampus (Schwenk et al., 2009) we found it also in the vast majority of NG2 glial cells. This subunit regulates trafficking of the receptors, slows channel gating and increases CTZ potency in cells co-expressing TARP γ8 (Schwenk et al., 2009; Shi et al., 2010; Gill et al., 2012). Expression of TARPs and CNIH-2 can decrease the polyamine block (Coombs et al., 2012). The latter study has also found CNIH-2 protein in cultured optic nerve oligodendroglial precursor cells. In heterologous expression systems, TARPs γ4, 7, and 8 mediate a recovery from desensitization called resensitization, which is abrogated by CNIH-2 (Kato et al., 2010). The lack of resensitization in NG2 glia (Seifert and Steinhäuser, 1995; Seifert et al., 1997b) might thus be due to abundant expression of CNIH-2 as demonstrated in the present study.

### Pharmacological Determination of Ca^2+^ Permeability of Glial AMPA Receptors

To further characterize the Ca^2+^ permeability of AMPA receptors in NG2 glia, polyamine analogs were used, which recognize binding sites within the pore. Transcript expression of GluA1-4 did not change during postnatal development, with GluA2 being the most abundant subunit. GluA2 limits the Ca^2+^ permeability of the receptors and prevents polyamine binding (Bowie and Mayer, 1995; Washburn et al., 1997). TARPs and CNIH2 also attenuate polyamine block of Ca^2+^ permeable receptors (Coombs et al., 2012; Soto et al., 2014). Expression of the latter might have prevented the polyamine block in our experiments.

In NG2 glia of mice early after birth (up to p5), we observed inward rectifying receptor currents in the presence of intracellular spermine, indicating expression of Ca^2+^ permeable receptors, which disappeared by p10 (Seifert et al., 2003). In rat, however, spermine sensitivity was still observed 2 weeks postnatally (Bergles et al., 2000). Naspm and intracellular spermine also inhibited EPSCs in NG2 glia of rat hippocampus, and the efficiency of inhibition declined with increasing age (Ge et al., 2006) while in mice, we observed no developmental changes. This species difference might be due to different contributions of TARPs/CNIHs to the glial AMPA receptor complexes. The latter study also revealed a physiological significance of AMPA-receptor-mediated Ca^2+^ influx, by demonstrating long-term potentiation at neuron-NG2 glia synapses at early developmental stages.

A Ca^2+^ permeability of AMPA receptors in juvenile NG2 glia has already been demonstrated with Ca^2+^ imaging (Jabs et al., 1994). In the present study, the inward rectifying I/V relations of Naspm- and IEM-1460-sensitive currents and analyses in solutions with Ca^2+^ as the sole charge carrier confirmed functional expression of Ca^2+^ permeable receptors. Masking of intracellular spermine block of outward currents through Ca^2+^ permeable AMPA receptors, as observed here, was previously demonstrated in heterologous expression systems and cultured cortical glial cells (Meucci and Miller, 1998), and might hint at variable contributions of GluA2 to the tetrameric channels [reviewed by Bowie (2012)]. The increased extracellular polyamine block of receptor currents at negative voltages, we have observed at p60, indicated enhanced expression of Ca^2+^ permeable AMPA receptors in mature NG2 glia. A recent publication demonstrated that intracellular Naspm confers complete and TARP-independent block to Ca^2+^ permeable, GluA2-lacking AMPA receptors (Coombs et al., 2021). In our study, intracellular Naspm led to inwardly rectifying I/V relations of AMPA receptor responses in 8/18 NG2 glia at p60 while at p10, 17/18 cells showed outward rectification. The inhibitory effect of Naspm and IEM-1460 on synaptic currents did not change during development. Block of evoked synaptic responses may have indicated that Ca^2+^ permeable AMPA receptors were located on postsynaptic glial membranes (Lujan et al., 2019).

Some anti-epileptic drugs, for example perampanel (Chappell et al., 2002; French et al., 2012), target AMPA receptors. However, AMPA receptor blockers harbor many undesired side effects, because of their unspecific dampening of excitation throughout the brain. The relative brain region- and/or cell type-specific expression of TARPs, for example TARP γ2 in the cerebellum, TARP γ8 in the hippocampus, TARP γ4 in glial cells, might allow for the development of new drugs with better specificity. To modulate excitatory transmission in the hippocampus, the TARP γ8 specific antagonists LY3130481 and JNJ-55511118 were developed, which leave AMPA receptor complexes lacking this auxiliary subunit unaffected (Gardinier et al., 2016; Maher et al., 2016). These antagonists inhibit excitation in the cortex and hippocampus, dampen cortical EEG activity and have anticonvulsant effects, with only mild motor and learning deficits (Kato et al., 2016; Maher et al., 2016). In our study, TARP γ8 was downregulated during maturation, as evidenced by its lower incidence in both single cells and after bulk isolation. Unexpectedly, however, modulation by JNJ was more pronounced in NG2 glia from older mice. This result might be explained by interactions of different TARPs/CNIH-2 with the receptor complex (Schober et al., 2011).

## Conclusion

NG2 cells in hippocampus primarily express the TARP subunits γ4, γ7, and γ8 as well as CNIH-2. These auxiliary subunits may slow receptor desensitization, increase kainate efficacy and reduce the sensitivity of Ca^2+^ permeable AMPA receptors to endogenous polyamines. Ca^2+^ influx through somatic AMPA receptors may regulate proliferation and differentiation (Chen et al., 2018), and deletion of AMPA receptors in NG2 glia reduces the survival of oligodendrocytes (Kougioumtzidou et al., 2017). Systematic analysis of mice with deletions of AMPA receptor subunits in NG2 glia may help to decipher its specific role in neural signaling.

## Data Availability Statement

The original contributions presented in the study are included in the article/Supplementary Material, further inquiries can be directed to the corresponding author/s.

## Ethics Statement

Ethical review and approval was not required for the animal study because we only performed organ harvesting after anesthesia.

## Author Contributions

CS and GS designed and supervised the experiments. SH, DT, SP, AT, RJ, and GS performed and/or analyzed the experiments. SH, GS, and CS wrote the manuscript. All authors contributed to the article and approved the submitted version.

## Conflict of Interest

The authors declare that the research was conducted in the absence of any commercial or financial relationships that could be construed as a potential conflict of interest.

## Footnotes

1 http://www.R-project.org

## Supplementary Material

Supplementary Figure 1 Analysis of GluA subunit expression in FAC sorted NG2 glia. Gene expression ratios, GluA/β-actin, were determined by qPCR according to equation (1). Data represent mean ± SD (juvenile, blue, *n* = 10; adult, red *n* = 10). The ratios did not change during development.

Supplementary Figure 2 Inhibition of AMPA receptor currents in freshly isolated NG2 glial cells by Naspm (50 μM) and JNJ (1 μM). **(A)** Membrane currents were elicited as described in Figures 2, 4 after adding quinine (100 μM) and BaCl_2_ (100 μM) to the bath solution. Responses were evoked by co-applying kainate (250 μM) with CTZ (100 μM), and inhibited by JNJ and JNJ + Naspm as indicated. **(C)** Averaged relative fractions of receptor currents sensitive to 1 μM JNJ and 50 μM Naspm (p10: *n* = 9; p60: *n* = 9). For comparison, the data obtained with 10 μM JNJ (Figures 4F, 5F) were included.

Supplementary Table 1 Primers used for single cell RT-PCR.
