# Supplementary figures and images for: Auxiliary Subunits Control Function and Subcellular Distribution of AMPA Receptor Complexes in NG2 Glia of the Developing Hippocampus
Source: Front Cell Neurosci. 2021 Jun 10;15:669717. doi: 10.3389/fncel.2021.669717 (PMC8222826; doi:10.3389/fncel.2021.669717)

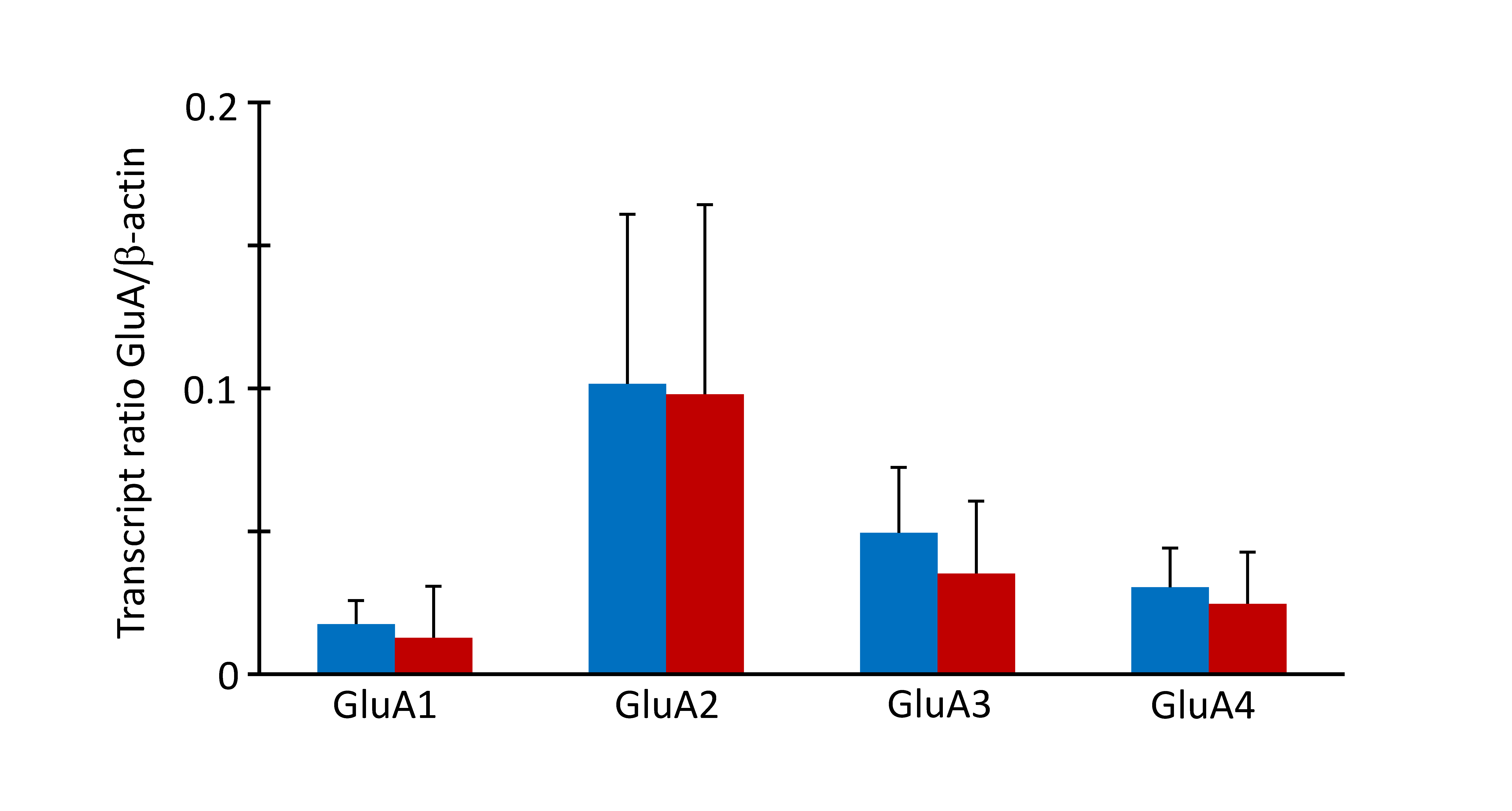

Supplement: Supplementary Figure 1 — Analysis of GluA subunit expression in FAC sorted NG2 glia. Gene expression ratios, GluA/β-actin, were determined by qPCR according to equation (1). Data represent mean ± SD (juvenile, blue, n = 10; adult, red n = 10). The ratios did not change during development. [file Image_1.TIF]

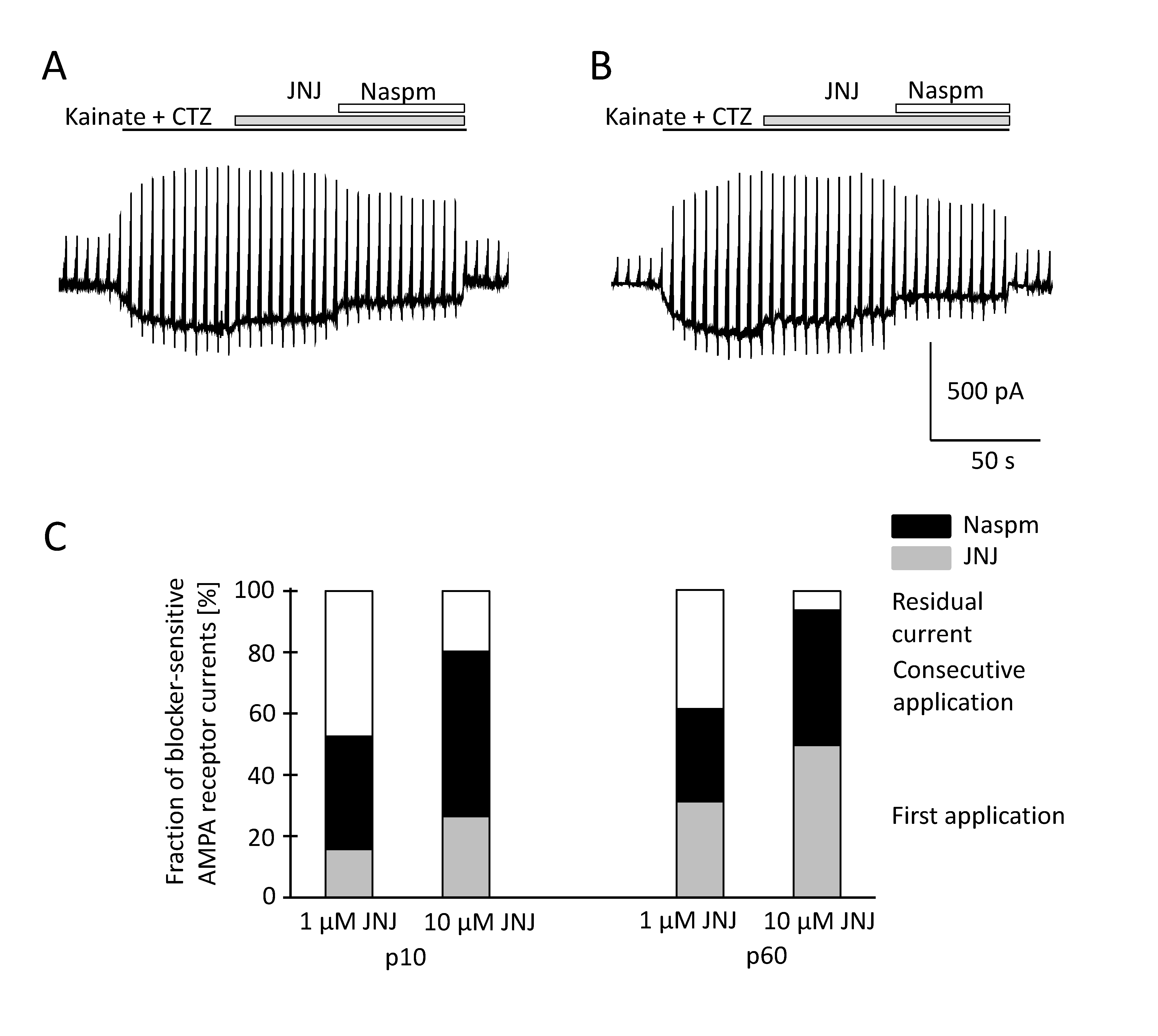

Supplement: Supplementary Figure 2 — Inhibition of AMPA receptor currents in freshly isolated NG2 glial cells by Naspm (50 μM) and JNJ (1 μM). (A) Membrane currents were elicited as described in Figures 2, 4 after adding quinine (100 μM) and BaCl2 (100 μM) to the bath solution. Responses were evoked by co-applying kainate (250 μM) with CTZ (100 μM), and inhibited by JNJ and JNJ + Naspm as indicated. (C) Averaged relative fractions of receptor currents sensitive to 1 μM JNJ and 50 μM Naspm (p10: n = 9; p60: n = 9). For comparison, the data obtained with 10 μM JNJ (Figures 4F, 5F) were included. [file Image_2.TIFF]
