# Supplementary material for: Auxiliary Subunits Control Function and Subcellular Distribution of AMPA Receptor Complexes in NG2 Glia of the Developing Hippocampus
Source: Front Cell Neurosci. 2021 Jun 10;15:669717. doi: 10.3389/fncel.2021.669717 (PMC8222826; doi:10.3389/fncel.2021.669717)
Supplement: Supplementary Table 1 — Primers used for single cell RT-PCR. [file Table_1.doc]

Supplemental Table 1: Primers used for single cell RT-PCR

| Gene | Sequence | Position | Product length | Accession No. |
| --- | --- | --- | --- | --- |
| TARP g2/3 | se 5‘-CCGGAAGATGCGGACTAYGAA  as 5‘-GGGGTCTCCAGCATTGGCT | 250, 250  474, 474 | 243 bp |  |
| TARP g-2 (nested) | se 5‘-GCTGACACCGCAGAGTATTTCCTC  as 5‘-CGATGATCCCGATGATATTACTAA | 271  440 | 193 bp | NM_007583 |
| TARP g-3 (nested) | se 5‘-AGGGCCTCCAGCGTCTTTC  as 5’-TATGATGCCGATGATGTTGCTTAG | 304  439 | 159 bp | NM_019430 |
| TARP g4/8 | se 5’-ATCGCCATCGGCACTGACTACTGG  as 5’-AGGCCTGCTGCCACAAASAGGAT | 76, 109  427, 487 | 374, 401 bp |  |
| TARP g4  (nested) | se 5’-GGGCACTGCTTCCGGATCAACCAC  as 5’-GCCGATGCAGAGCCCTCCGAGCAG | 232  355 | 147 bp | NM_019431 |
| TARP g8  (nested) | se 5’-GGGCAGTGGCTCCTCCGAGAAGA  as 5’-ACTCCGCGCTGTCGTGGTCGTAGT | 204  332 | 152 bp | NM_133190 |
| TARP g5/7 | se 5’-AGCAACATCGGCCACATCC  as 5’-CTGGCCGGAGTAATCGGAGCAGT | 352, 352  668, 668 | 339 bp |  |
| TARP g5  (nested) | se 5’-CAAGGATGCAGAGACCTATTTCA  as 5’-GGCCGGTAGAAGCCTGGGTGAG | 492  635 | 165 bp | NM_080644 |
| TARP g7  (nested) | se 5’-GCAGCTCTGAGCAGTACTTTCACT  as 5’-GCTGAGACGCGGTCGGTAGAAGG | 494  644 | 173 bp | NM_133189 |
| CNIH2/3 | se 5’-GGTGCTGTGCGCTKCSCTCATCTT  as 5’-GGACAGGAGGTAGAAGGCSAGYTT | 42, 42  412, 469 | 394, 451 bp |  |
| CNIH2  (nested) | se 5’-CGGGCACGCGAGCGTTTGAAA  as 5’-GAGACCGCATCATACATGACCTCA | 145  339 | 218 bp | NM_009920 |
| CNIH3  (nested) | se 5’-CCCCAGGTGGACGGAGGAAG  as 5’-GCTCTGAGCTATCTGCTGGACAGT | 212  377 | 189 bp | NM_028408 |
| PDGFRα | se 5’-TCAAAGGGAGGACGTTCAAGACC | 578 | 303 bp | NM_011058 |
|  | as 5’-GACGGGCAGCACATTCATACTC | 859 |  |  |
| PDGFRα | se 5’-TGTGTATAAGGCAGGAGAAACGAT | 669 | 167 bp |  |
| (nested) | as 5’-TGGGGACGGTCAAAGTGTA | 817 |  |  |

Position 1 is the first nucleotide of the initiation codon. The length of PCR products was indicated as base pairs (bp). ‘Se’ and ‘as’ mark sense and antisense primers. All sense and antisense primers are located on different exons, respectively.
